# Predictive Value of a Profile of Routine Blood Measurements on Mortality in Older Persons in the General Population: The Leiden 85-Plus Study

**DOI:** 10.1371/journal.pone.0058050

**Published:** 2013-03-04

**Authors:** Anne H. van Houwelingen, Wendy P.J. den Elzen, Simon P. Mooijaart, Margot Heijmans, Jeanet W. Blom, Anton J. M. de Craen, Jacobijn Gussekloo

**Affiliations:** 1 Department of Public Health and Primary Care, Leiden University Medical Center, Leiden, the Netherlands; 2 Department of Gerontology and Geriatrics, Leiden University Medical Center, Leiden, the Netherlands; 3 Institute for Evidence-Based Medicine in Old Age (IEMO), Leiden, the Netherlands; Cardiff University, United Kingdom

## Abstract

**Background:**

Various questionnaires and performance tests predict mortality in older people. However, most are heterogeneous, laborious and a validated consensus index is not available yet. Since most older people are regularly monitored by laboratory tests, we compared the predictive value of a profile of seven routine laboratory measurements on mortality in older persons in the general population with other predictors of mortality; gait speed and disability in instrumental activities of daily living (IADL).

**Methodology/Principal Findings:**

Within the Leiden 85-plus Study, a prospective population-based study, we followed 562 participants aged 85 years for mortality over five years. At baseline (age 85 years) high-density lipoprotein cholesterol, albumin, alanine transaminase, hemoglobin, creatinin clearance, C-reactive protein and homocysteine were measured. Participants were stratified based on their number of laboratory abnormalities (0, 1, 2–4 and 5–7). The predictive capacity was compared with gait speed (6-meter walking test) and disability in IADL (Groningen Activity Restriction Scale) by C-statistics. At baseline, 418 (74%) 85-year old participants had at least one laboratory abnormality. All cause mortality risk increased with increasing number of laboratory abnormalities to a hazard ratio of 5.64 [95% CI 3.49–9.12] for those with 5–7 laboratory abnormalities (p<0.001) compared to those without abnormalities. The c-statistic was 0.66 [95% CI 0.59–0.69], similar to that of gait speed and disability in IADL.

**Conclusions/Significance:**

In the general population of oldest old, the number of abnormalities in seven routine laboratory measurements predicts five-year mortality as accurately as gait speed and IADL disability.

## Introduction

Prognostic information about life expectancy in older people is important in clinical decision-making because this population is very heterogeneous. Whereas for vital older people usual care is recommended, older people with a limited life expectancy may benefit from integrated, pro-active care [1]. In addition, although older people with a better life expectancy may benefit from cancer screening or advanced medical techniques [2], it is questionable whether older people with a higher mortality risk should be exposed to such invasive tests and/or treatments.

Various prognostic indices are available to predict prognosis in older people [3]. Self-reported questionnaires and performance tests are often used to identify older people at risk for mortality [4]–[6]. However, these instruments are heterogeneous and a consensus index is not yet available [4]–[6]. Moreover, the application of self-reported questionnaires and performance tests in the general population is laborious and time consuming. Arguably, health care for older people in general could be improved by the development of a robust prognostic tool that is easy to use, inexpensive, fast, and not dependent on healthcare personnel.

Routine clinical laboratory measurements may provide such a clinical prognostic tool. Since most older people are regularly monitored by laboratory tests for preventive or disease-related purposes, such tests may provide valuable prognostic information about older persons. Several common abnormal laboratory results are known to be predictive of poor outcomes in older persons, such as high C-reactive protein (CRP) level [7], high homocysteine level [8], low high-density lipoprotein-cholesterol (HDL-C) [9], low albumin level [10], low alanine transaminase level [11], low hemoglobin level [12], [13], and poor kidney function (low creatinin clearance) [14]. Moreover, combining laboratory results into a laboratory prognostic index maximizes their predictive utility [15]–[20].

Therefore, this study examines whether and to what extent a profile of seven routine laboratory parameters can predict mortality in persons aged 85 years. In addition, the results from this profile are compared with other known predictors of mortality, i.e. gait speed [21] and disability in instrumental activities of daily living (IADL) [22].

## Methods

### Study Population

This study was performed within the Leiden 85-plus Study, a population-based prospective follow-up study of 85-year-old inhabitants of the city of Leiden, the Netherlands [23]. Between September 1997 and September 1999, 705 inhabitants of Leiden reached the age of 85 years and were eligible to participate in this study. No exclusion criteria were applied. Fourteen persons died prior to enrolment and 92 refused participation; 7 persons died before blood sample collection and 30 refused blood sampling. As a result, baseline laboratory data for 562 participants (80% of the eligible patients) were available for this study.

At age 85, participants were visited at their place of residence. During these visits, participants underwent face-to-face interviews and were weighed. In addition, performance tests were done and a venous blood sample was drawn. Information on medical history was obtained from standardised interviews with the participant’s general practitioner (GP) or treating elderly care physician (for participants living in a nursing home). All participants gave written informed consent for the study including the use of data from their medical records for additional analysis, following explanation of the study requirements and assurance of confidentiality and anonymity. For participants with severe cognitive impairment, written informed consent was obtained from a proxy. The Medical Ethical Committee of the Leiden University Medical Center approved the study and the informed consent procedure.

### Study Parameters

#### Laboratory profile

For each individual participant, we composed a profile of results of seven laboratory measurements: CRP, homocysteine, hemoglobin, HDL-C, alanine transaminase, albumin, and creatinin clearance. These seven laboratory abnormalities were included as markers of different physiological systems; general health status (CRP), cardiovascular status (homocysteine), hematological status (hemoglobin), fatty acid metabolism (HDL-C), liver function (alanine transaminase), nutritional status (albumin), and renal function (creatinin clearance).

Non-fasting blood plasma samples were drawn before 11 am. All samples arrived within 2 hours after the sample was drawn at the laboratory. Hemoglobin levels were then determined on the day the sample was drawn with the use of an automated clinical analyzing system (Coulter Electronics, Hialeah, Florida, USA). After centrifuging with citrate as anticoagulant, plasma samples were frozen immediately to measure concentrations of homocysteine later in one batch in frozen plasma samples with a fluorescence polarization immunoassay after reduction to the free form with an IMx analyzer (Abbott, Abott Park, IL, USA) (coefficient variation 2.2–2.5%). HDL-C, albumin, alanine transaminase, creatinin, and CRP were determined on the day the sample was drawn using the fully automated Hitachi 747 and 911 (Hitachi, Tokyo, Japan). Creatinin clearance was estimated with the Cockcroft Gault formula [24].

Since clinical cut-offs are reported to be unreliable in old age [25], [26], each parameter was ranked in sex-dependent quartiles. With univariate Cox proportional hazard models, we determined which sex-dependent quartile (high or low) per laboratory measurement predicted the highest mortality risk. Per participant, the number of abnormal laboratory results was summed, i.e. the highest quartile of CRP and homocysteine, and the lowest quartile of hemoglobin, HDL-C, alanine transaminase, albumin, and creatinin clearance.

Scores ranged from zero laboratory abnormalities to seven laboratory abnormalities. Participants were stratified in four risk groups based on the number of abnormalities (no laboratory abnormality, 1 laboratory abnormality, 2–4 laboratory abnormalities and 5–7 laboratory abnormalities).

#### Gait speed

Gait speed was assessed at the participant’s home with a 12-m walking test, which is described in detail elsewhere [27]. In short, the course was denoted by a tape measurement of 3 m. Participants were requested to walk 2 times back and forth along the tape as quickly as possible, from a standing start position. Use of a walking aid was allowed. Total time was measured with a stopwatch. For this study we used 6-m gait speed, which is the time an older person needed for one time back and forth along a 3 meter long tape. Gait speed was calculated using distance in meters and time in seconds (m/s) for 497 (88.4%) participants. A total of 65 participants were unable to perform this test. Since older people who are unable to walk are at highest risk, those participants were considered as having the lowest possible gait speed. Gait speed was ranked in four risk groups based on sex-dependent quartiles.

#### Ability in instrumental activities of daily living

Disability in Instrumental Activities of daily Living (IADL) was measured annually with the Groningen Activities Restriction Scale (GARS) [28]. The GARS assesses restrictions in competence in nine basic activities of daily living (BADL) and nine IADL items. It is a self-report questionnaire and assesses therefore if someone can do the task, not if someone actually performs the task [29]. For the present analyses, only the IADL items were included. IADL included the following tasks: doing light housework, heavy cleaning, wash and iron clothes, clean and make the bed, prepare a hot meal, climbing stairs, get around outdoors, do the groceries, and attend to feet and toenails. Questions are phrased: ‘*Can you fully independently*,…?’ Answers range from ‘fully independently, without any difficulty’ (1 point) to ‘not fully independently with someone’s help’ (4 points).

A summed score for IADL was calculated ranging from 9 (indicating ability to perform all activities without assistance) to 36 (indicating disability). The summed score of all participants was ranked in sex-dependent quartiles.

#### Mortality

Mortality data, recorded from the start of the study until participants reached the age of 90 years, were obtained from the municipal registry. Causes of death were obtained from Statistics Netherlands (CBS), where all national death certificates are coded according to the International Classification of Diseases and Related Disorders, 10th revision. Causes of death were divided into cardiovascular causes (codes I00–I99) and non-cardiovascular causes (all codes except I00–I99) [30].

#### Other parameters

Information on sex, level of education and institutionalization was obtained during face-to-face interviews with participants. Level of education was measured as the highest educational degree the participant had obtained. Cognitive function was measured annually with the Mini-Mental State Examination (MMSE) [31]; scores range from 0–30 points, with lower scores indicating poorer cognitive performance. Multimorbidity was defined as the presence of one or more diseases at baseline as indicated by the participants’ GP, elderly care physicians, pharmacy records and laboratory findings, and included stroke, myocardial infarction, severe cognitive impairment, diabetes mellitus, Parkinson disease, hip fracture, arthritis, obstructive lung disease, and cancer [32]. The presence of severe cognitive impairment was based on a diagnosis by the participant’s treating physician or a MMSE score <19 points [33]. The presence of diabetes was based on a diagnosis by the treating physician, a non-fasting glucose level >200.0 mg/dL, or the use of anti-diabetic medication.

### Statistical Analysis

Baseline differences between participants in the four risk groups of the laboratory profile were compared with the Jonckheere Terpstra test (for continuous nonparametric variables) or linear by linear test (for categorical variables). Kaplan-Meier curves (including log rank tests) and Cox proportional hazard models were used for the prediction of the three models (laboratory profile, gait speed, and IADL) on mortality. Since the aim of this study was to assess the predictive performance of the laboratory profile, and not to investigate the causes of disease, no adjustments were made for potential confounders.

We assessed the performance of the different prediction models with receiver operation characteristic (ROC) curves with corresponding c-statistics (neutral value 0.50 and 95% confidence intervals (CI)), using all-cause mortality as the outcome.

As additional sensitivity analyses, stratified analyses were performed for the presence of multimorbidity at baseline.

Data were analyzed with Predictive Analytics SoftWare 17.0 for Windows. A p-value of <0.05 was considered statistically significant. The reporting of this observational study followed guidelines from the STROBE statement [34].

## Results

Table 1 shows the characteristics of the total study population, stratified for the four risk groups of the laboratory profile. Of the 562 participants, 373 (66.4%) were female and 102 (18.1%) were living in a home for the elderly or in a nursing home.

**Table 1:** Baseline characteristics of the study population at age 85 years stratified according to the number of abnormal laboratory results.

	All	Number of abnormal laboratory results at baseline				p for trend
		0	1	2–4	5–7	
	n = 562	n = 144	n = 165	n = 216	n = 37	
Female	373 (66.4)	97 (67.4)	107 (64.8)	142 (65.7)	27 (73.0)	0.042
Low level of education (primary school only)	363 (64.4)	89 (61.8)	94 (57.0)	164 (71.3)	26 (70.3)	0.013
Low income <€750 monthly	284 (50.5)	65 (45.1)	75 (45.5)	124 (57.4)	20 (54.1)	0.01
Home for the elderly/nursing home	102 (18.1)	14 (9.7)	22 (13.3)	50 (23.1)	16 (43.2)	<0.01
≥1 chronic diseases*	420 (74.7)	91 (63.2)	126 (76.4)	173 (80.1)	30 (81.1)	<0.01
Mini-mental state examination score (points)	26 (22–28)	27 (24–29)	27 (24–49)	25 (19–28)	22 (17–27)	<0.01
Disability in instrumental activities of daily living score (pts)	18 (12–26)	15 (11–21)	17 (12–25)	21 (14–31)	22 (17–27)	<0.01
6-meter gait speed (m/s)	1.9 (1.5–2.8)	1.8 (1.4–2.4)	1.9 (1.5–2.6)	2.1 (1.5–3.1)	2.3 (1.8–3.8)	<0.01

Continuous data are presented as median (IQR); p for trend values were obtained by Jonckheere Terpstra tests.

Categorical data are presented as number (%); p for trend values were obtained by Linear by Linear tests.

* cancer, myocardial infarction, stroke, dementia, diabetes, chronic obstructive pulmonary disease, Parkinson’s disease, hip fracture, arthritis.

At baseline, 144 participants (26.0%) had 0 abnormal laboratory results, 165 (29.4%) had 1 abnormal laboratory result, 216 participants (38.4%) had 2, 3 or 4 abnormal laboratory results, and 37 participants (6.6%) had 5, 6 or 7 abnormal laboratory results. All combinations of laboratory abnormalities for participants with 2 or 3 abnormalities occurred in a similar frequency (data not shown).

With an increasing number of abnormal laboratory results, participants were more likely to have a low income and to live in a home for the elderly or in a nursing home. In addition, participants with an increasing number of abnormalities had more multimorbidity, more disability in IADL, lower gait speed, and lower MMSE scores (Table 1).

During the 5-year follow-up, 260/562 (46%) participants died. In the univariate analysis, participants with levels within the highest quartile of CRP and homocysteine, and within the lowest quartile of hemoglobin, HDL-C, alanine transaminase, albumin and creatinin clearance, had the highest all-cause mortality risk compared to participants within the other quartiles of these laboratory values (Table 2; all p-trend <0.005, all p for 4^th^ quartile compared to other quartiles combined <0.005).

**Table 2:** Univariate all-cause mortality risks for sex-dependent quartiles of laboratory results included in the laboratory profile (n = 562).

	Quartile of laboratory results				p for trend	P for 4th quartile compared to the other 3 quartilescombined
	1	2	3	4		
C-reactive protein	1 (ref)	1.10 (0.75–1.61)	1.36 (0.96–1.94)	2.11 (1.51–2.95	<0.001	<0.001
Homocysteine	1 (ref)	1.14 (0.76–1.71)	2.04 (1.40–2.60)	2.75 (1.92–3.96)	<0.001	<0.001
Hemoglobin*	1 (ref)	0.98 (0.67–1.42)	1.04 (0.72–1.48)	1.82 (1.31–2.54)	<0.001	<0.001
High density lipoprotein cholesterol*	1 (ref)	1.04 (0.72–1.50)	1.01 (0.70–1.47)	1.86 (1.33–2.60)	<0.001	<0.001
Alanine transaminase*	1 (ref)	1.00 (0.69–1.44)	0.88 (0.61–1.26)	1.72 (1.23–2.39)	0.005	0.004
Albumin*	1 (ref)	1.41 (0.94–2.19)	2.10 (1.45–3.06)	3.39 (2.33–4.95)	<0.001	<0.001
Creatinin clearance*	1 (ref)	0.96 (0.66–1.41)	1.32 (0.92–1.89)	1.85 (1.31–2.61)	<0.001	<0.001

Data represent hazard ratios and 95% confidence intervals, calculated with the univariate Cox-proportional hazard model.

Laboratory results are divided into sex-dependent quartiles.

25th, 50th and 75th percentile limits of laboratory results stratified for sex:

Hemoglobin: male 12.5–13.4–14.2 g/dL; female 12.0–12.8–13.6 g/dL.

High-density lipoprotein cholesterol: male 35.5–42.5–51.7 mg/dL; female: 41.7–52.1–61.8 mg/dL.

Alanine transaminase: male 11–15–20 U/L; female: 11–14–17 U/L.

Albumin: male: 4.0–4.2–4.4 g/dL; female: 4.0–4.2–4.4 g/dL.

Creatinin clearance: male: 39.4–47.2–53.9 ml/min; female: 36.8–43.4–50.8 ml/min.

C-reactive protein: male: 2–4–8 mg/L; female: 1–4–8 mg/L.

Homocysteine: male: 1.47–19.8–25.6 mg/L; female: 15.0–17.9–22.8 mg/L.

* highest to lowest quartile.

**Figure 1:**
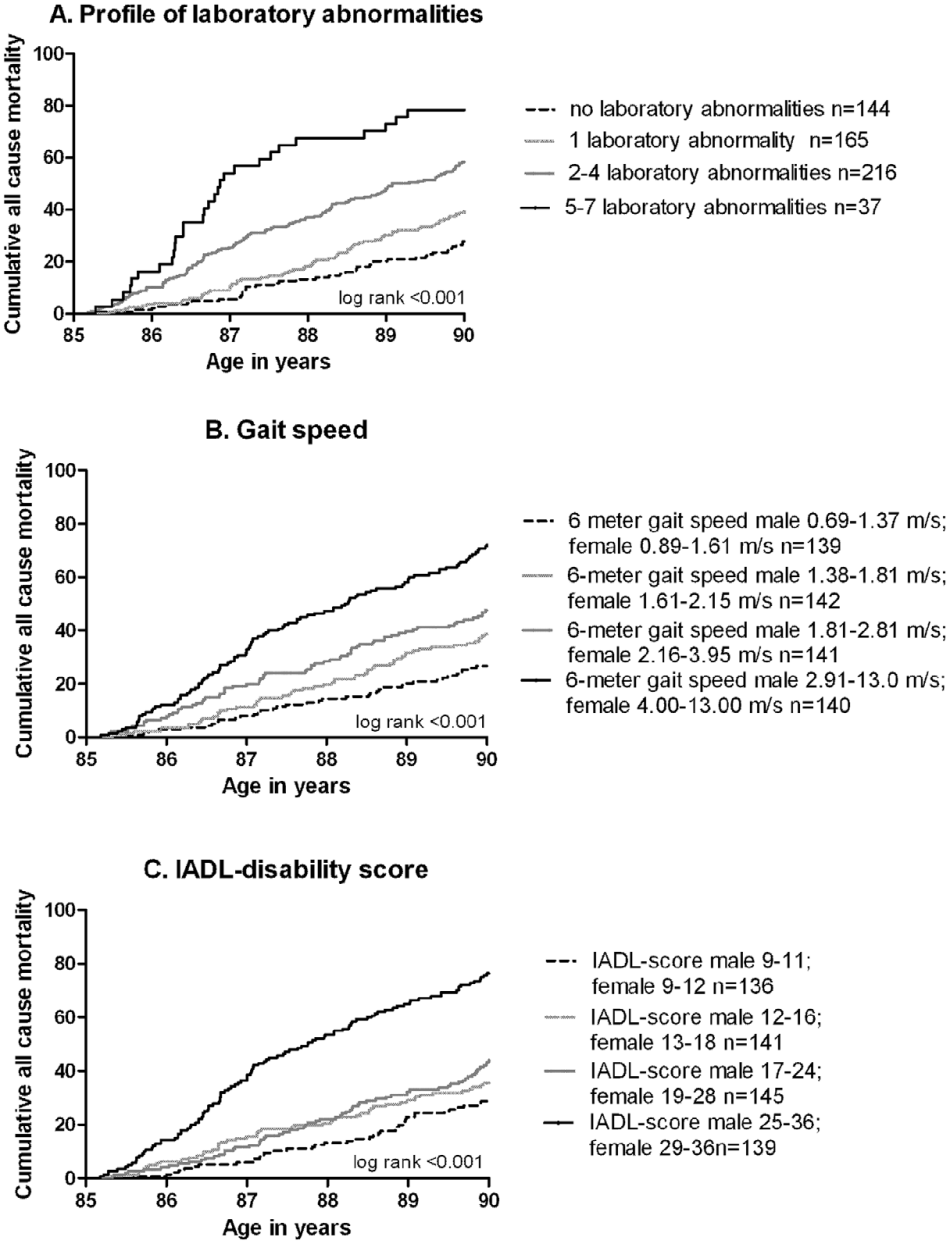
Kaplan Meier cumulative mortality curves for all cause mortality according to the three models. (A) laboratory profile based on sex specific quartiles of the seven included laboratory values, (B) sex specific quartiles of gait speed and (C) sex specific quartiles of instrumental activities of daily living (IADL) at age 85 years. **A** **- - -** no laboratory abnormalities n = 144, **-----**1 laboratory abnormality n = 165, **-----**2–4 laboratory abnormalities n = 216, **-----**5–7 laboratory abnormalities n = 37. **B - - -** 6-meter gait speed male 0.69 –1.37 m/s; female 0.89–1.61 m/s n = 139, **-----** 6-meter gait speed male 1.38–1.81 m/s; female 1.61–2.15 m/s n = 142, **-----** 6-meter gait speed male 1.81–2.81 m/s; female 2.16–3.95 n = 141, **-----**6-meter gait speed male 2.91–13.0 m/s; female 4.00–13.00 m/s n = 140. **C - - -** IADL-score male 9–11; female 9–12 n = 136, **-----** IADL-score male 12–16; female 13–18 n = 141, **-----** IADL-score male 17–24; female 19–28 n = 145, **-----** IADL-score male 25–36; female 29–36 n = 139.

Figure 1 presents the cumulative mortality curves for all-cause mortality in the four risk groups depending on the laboratory profile (panel A), gait speed (panel B) and ability in IADL (panel C). Participants with 5–7 laboratory abnormalities, participants in the lowest gait speed group, and participants with the highest disability in IADL had the highest all-cause mortality risk (all log rank tests p<0.001).

Table 3 shows all-cause and cause-specific mortality risks for participants in the four risk groups based on the laboratory profile, the 6-m gait speed and the summed IADL score. Participants with 5–7 abnormal laboratory results had the highest all-cause mortality risk, compared to participants with 0 laboratory abnormal laboratory results (hazard ratio [HR] 5.64, 95% CI 3.49–9.12) (p-trend <0.001). Per additional laboratory abnormality, the mortality risk increased 1.38-fold (HR 1.38, 95% CI 1.29–1.49) (p-trend <0.001).

**Table 3:** All-cause and cause-specific absolute and relative mortality risk stratified for sex-dependent quartiles of the laboratory profile, IADL disability score and 6-meter gait speed.

	Risk group of predictor												
	1			2			3			4			
	Absolute risk/100 py	Hazard ratio (95% CI)		Absolute risk/100 py	Hazard ratio (95% CI)		Absolute risk/100py	Hazard ratio (95% CI)		Absolute risk/100py	Hazard ratio(95% CI)		p for trend
**Laboratory profile**													
All-cause mortality	6.23	1 (ref)		9.38	1.52 (1.03–2.26)		16.60	2.79 (1.96–3.99)		30.94	5.64 (3.49–9.12)		<0.001
Cardiovascular mortality	3.12	1 (ref)		2.89	0.93 (0.50–1.73)		6.32	2.08 (1.23–3.50)		14.93	5.17 (2.60–10.27)		<0.001
Non-cardiovascular mortality	3.12	1 (ref)		6.50	2.13 (1.26–3.61)		10.27	2.22 (1.59–3.09)		16.00	3.79 (2.16–6.67)		<0.001
**Gait speed**													
All-cause mortality	6.00	1 (ref)		9.29	1.57 (1.03–2.38)		13.74	2.13 (1.43–3.19)		22.92	4.13 (2.83–6.03)		<0.001
Cardiovascular mortality	2.43	1 (ref)		3.72	1.53 (0.80–2.96)		5.20	2.16 (1.15–4.05)		8.39	3.57 (1.96–6.52)		<0.001
Non-cardiovascular mortality	3.57	1 (ref)		5.58	1.59 (0.93–2.74)		7.24	2.11 (1.25–3.56)		14.52	4.53 (2.78–7.36)		<0.001
**IADL disability**													
All-cause mortality	6.46	1 (ref)		9.82	1.35 (0.89–2.05)		10.77	1.70 (1.14–2.53)		25.94	4.43 (3.07–6.42)		<0.001
Cardiovascular mortality	3.31	1 (ref)		3.10	0.94 (0.50–1.79)		3.53	1.07 (0.58–1.99)		10.28	3.28 (1.91–5.60)		<0.001
Non-cardiovascular mortality	3.15	1 (ref)		5.51	1.77 (1.00–3.12)		7.24	2.35 (1.37–4.03)		15.66	5.66 (3.38–9.46)		<0.001

Abbreviations: CI, confidence interval; IADL, instrumental activities of daily living.

Gait speed and IADL disability were divided into sex-dependent quartiles.

25th, 50th and 75th percentile limits of laboratory measurements stratified for sex;

Gait speed: male 1.37–1.81–2.91 m/s; female 1.61–2.15–3.95 m/s.

IADL disability score: male 11–17–25 points; female 12–19–29 points.

Participants in the group with the lowest gait speed had the highest mortality risk (HR 4.13, 95% CI 2.83–6.03) compared to those with the highest gait speed. Participants with the most disability in IADL (i.e. highest quartile of the summed IADL score) also had the highest mortality risk (HR 4.43, 95% CI 3.07–6.42) compared to participants with the least IADL disability.

Of the 260 participants that died, 102 (39.2%) died from cardiovascular causes and 158 (60.8%) from non-cardiovascular causes. Similar associations of the laboratory profile, gait speed, and ability in IADL were observed for cardiovascular and non-cardiovascular mortality (Table 3).

The increased mortality risk for the risk groups based on the laboratory profile was most pronounced in older people with multimorbidity. In participants with multimorbidity the all-cause mortality risk for participants with 1 abnormality was HR 1.47 (95% CI 0.96–2.28), for participants with 2–4 abnormalities HR was 3.05 (95% CI 2.06–4.50), and for participants with 5–7 abnormalities HR was 8.71 (95% CI 5.03–15.06) (p-trend <0.001). Less pronounced results were found for participants without multimorbidity: for participants with 1 abnormality HR was 1.40 (95% CI 0.52–3.76), for 2–4 abnormalities HR was 1.90 (95% CI 0.81–4.47) and for 5–7 abnormalities HR was 2.14 (95% CI 0.55–8.28) (p-trend = 0.108).

Similar associations were found when using other classifications of our profile (quartiles, tertiles, three and seven risk groups) at baseline (data not shown).

**Figure 2:**
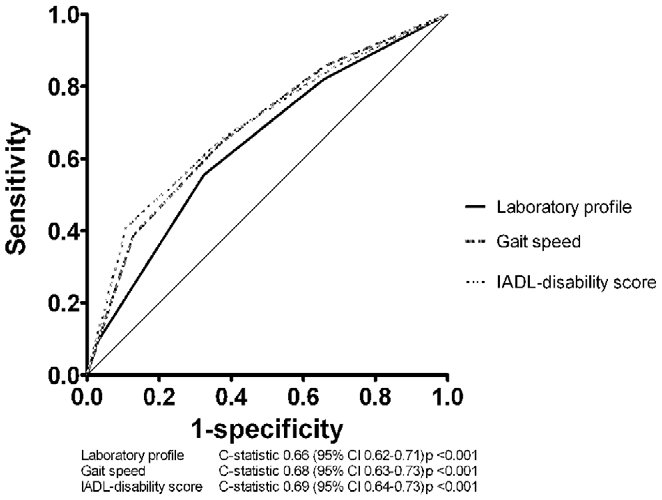
Performance of the three models for 5-year all-cause mortality in 562 participants aged 85 years. The three models were based on the profile of laboratory abnormalities, sex-dependent quartiles of gait speed and sex-dependent quartiles of the IADL-disability score. **-----** Laboratory profile **- - -** Gait speed **. . .** IADL-disability score.

When comparing the predictive value of the laboratory profile with the models on gait speed and ability in IADL with ROC curves (Figure 2), the c-statistic for the laboratory profile was 0.66 (95% CI 0.62–0.71), for the sex-dependent quartiles of 6-m gait speed it was 0.68 (95% CI 0.63–0.73) and for sex-dependent quartiles of ability in IADL it was 0.69 (95% CI 0.64–0.73).

## Discussion

In this population-based study of very old people, the number of abnormalities in a profile of seven routine laboratory measurements is a robust predictor of all-cause mortality. The predictive value of this laboratory profile was similar for cardiovascular and non-cardiovascular mortality. Moreover, mortality prediction by this laboratory profile was as accurate as models based on gait speed or IADL disability.

These results build on evidence from other studies. The present study confirms that abnormal levels of markers of physiological systems predict mortality in older individuals [19], [20], [35]–[38]. In contrast to these earlier studies we did not include non-laboratory markers of physiological systems (e.g. blood pressure), but selected seven common laboratory measurements reflecting dysregulation in one or more physiological systems. It is known that dysregulation in various physiological systems increases with age, and that dysregulation in multiple systems is associated with a higher mortality risk than dysregulation in one system alone [36], [37]. All of our seven routine laboratory measurements are reported to be individual predictors of poor outcome [7]–[14], and are individually used to guide clinical decisions and monitor disease in individual patients. This laboratory profile is easy to obtain and can be extracted from the biobanks of epidemiological studies, highlighting the potential value of this laboratory profile in older persons for research purposes.

Our study supports results from others showing that low gait speed is a powerful predictor of mortality in older people [21], and that a profile of seven routine laboratory measurements can predict mortality just as accurately. Measurement of gait speed requires training and time, and there is no uniform standard for the assessment of gait speed. Laboratory measurements are routinely determined when an older person is admitted to hospital and also in older people registered in a general practice. Therefore, this laboratory profile can be obtained with less effort than gait speed for assessing prognosis in both hospitalized and in community-dwelling older people.

Many other prognostic indices are available to predict all-cause mortality and to guide clinical decision-making [3], [39]. All these indices include chronological age, which is a powerful predictor of mortality, especially when combined with sex (c-statistic 0.75) [40]. In these models, additional predictors beyond age and sex only minimally increase discriminative power [40]. In the present study all participants were of the same age at baseline, and quartiles were sex-dependent. Although the predictive power of the laboratory profile is lower (c-statistic 0.66) compared to the earlier models, our model is valuable because it provides information beyond age and sex. In addition, it is known that biological age is more important in terms of prognosis than chronological age [41], [42]. Our profile might possibly be seen as an indicator of biological age and could, after validation in additional cohorts, be a useful tool for clinicians to assess biological age and prognosis.

The aim of this study was to investigate the predictive value of three models based on the laboratory profile, gait speed and IADL disability in the general population at large. Our study population therefore also included participants with severe cognitive impairment (MMSE <19, 90 participants). If a participant had severe cognitive impairment, a proxy was asked to attend the interview and, when necessary, to add information to the answers of the participants. Proxy information was used for IADL and BADL items of the GARS. Proxies may assess the situation of their relative either better or poorer than it actually was, resulting in non-selective misclassification of participants into incorrect IADL-quartiles. Our results may therefore be an underestimation of the true effect.

### Strengths and Limitations

Our study has several strengths. The Leiden 85-plus Study is a population-based prospective follow-up study with an 80% response rate and complete follow-up for mortality. These factors add to the external validity of our results. Whereas previous studies mainly focused on specific (younger) in-hospital populations, we have shown the predictive value of the laboratory profile in 85-year-old persons in the general population. Since the oldest old are the fastest growing segment of the general population, and our study is representative for this age group [23], these findings are particularly important. However, because the risk of common determinants of disease and mortality varies largely between age groups [25], [26] our results cannot automatically be extrapolated to younger populations. Another strength is that all the parameters were measured independently of adverse outcomes and without knowledge of the presence of disease. In addition, the use of quartiles of component measures instead of clinical cut-off points (that are still subject to debate), allowed us to interpret these data without prior assumptions.

A limitation of our study is the relatively small sample size. Because only 37 participants had 5, 6 or 7 abnormalities, this subgroup had to be combined. This was particularly true for older people without multimorbidity; however, trends in this group were similar to those with multimorbidity. Furthermore, we used only one baseline measurement of our laboratory profile; repeated measurements over time may provide additional information to stratify risk in this population. Another limitation is that the study was only used as a development cohort and no validation cohort was available to confirm the results. Also, since quartiles were used instead of clinical cut-off points, the use of our profile in other populations is not yet possible. Therefore, replication of these results in a validation cohort is necessary, also to establish absolute cut-offs for our index.

In conclusion, in this group of older persons, a laboratory profile of seven routine laboratory tests predicts mortality as accurately as models based on gait speed or IADL disability. This predictive study calls for confirmation in additional cohorts, as well as an in-depth analysis of the etiology and examination of its clinical use.

## References

[1] Reuben DB (2009) Medical care for the final years of life: “When you're 83, it's not going to be 20 years”. JAMA 302: 2686–2694. 302/24/2686 [pii];10.1001/jama.2009.1871 [doi]. 10.1001/jama.2009.1871 PMC2822435 20040557

[2] Balducci L (2007) Aging, frailty, and chemotherapy. Cancer Control 14: 7–12. 17242666 10.1177/107327480701400102

[3] Yourman LC, Lee SJ, Schonberg MA, Widera EW, Smith AK (2012) Prognostic indices for older adults: a systematic review. JAMA 307: 182–192. 307/2/182 [pii];10.1001/jama.2011.1966 [doi]. 10.1001/jama.2011.1966 PMC3792853 22235089

[4] Vermeulen J, Neyens JC, van Rossum E, Spreeuwenberg MD, de Witte LP (2011) Predicting ADL disability in community-dwelling elderly people using physical frailty indicators: a systematic review. BMC Geriatr 11: 33. 1471-2318-11-33 [pii];10.1186/1471-2318-11-33 [doi]. 10.1186/1471-2318-11-33 PMC3142492 21722355

[5] de Vries NM, Staal JB, van Ravensberg CD, Hobbelen JS, Olde Rikkert MG et al. (2011) Outcome instruments to measure frailty: a systematic review. Ageing Res Rev 10: 104–114. S1568-1637(10)00076-0 [pii];10.1016/j.arr.2010.09.001 [doi]. 10.1016/j.arr.2010.09.001 20850567

[6] Studenski S, Perera S, Wallace D, Chandler JM, Duncan PW, et al.. (2003) Physical performance measures in the clinical setting. J Am Geriatr Soc 51: 314–322. jgs51104 [pii]. 10.1046/j.1532-5415.2003.51104.x 12588574

[7] Willems JM, Trompet S, Blauw GJ, Westendorp RG, De Craen AJ (2010) White blood cell count and C-reactive protein are independent predictors of mortality in the oldest old. J Gerontol A Biol Sci Med Sci 65: 764–768. glq004 [pii];10.1093/gerona/glq004 [doi]. 10.1093/gerona/glq004 20106963

[8] de Ruijter W, Westendorp RG, Assendelft WJ, den Elzen WP, De Craen AJ, et al (2009) Use of Framingham risk score and new biomarkers to predict cardiovascular mortality in older people: population based observational cohort study. BMJ 338: a3083. 19131384 10.1136/bmj.a3083 PMC2615548

[9] Weverling-Rijnsburger AW, Jonkers IJ, Van Exel E, Gussekloo J, Westendorp RG (2003) High-density vs low-density lipoprotein cholesterol as the risk factor for coronary artery disease and stroke in old age. Arch Intern Med 163: 1549–1554. 10.1001/archinte.163.13.1549 [doi];163/13/1549 [pii]. 10.1001/archinte.163.13.1549 12860577

[10] Schalk BW, Visser M, Bremmer MA, Penninx BW, Bouter LM, et al. (2006) Change of serum albumin and risk of cardiovascular disease and all-cause mortality: Longitudinal Aging Study Amsterdam. Am J Epidemiol 164: 969–977. kwj312 [pii];10.1093/aje/kwj312 [doi]. 10.1093/aje/kwj312 16980573

[11] Ford I, Mooijaart SP, Lloyd S, Murray HM, Westendorp RG, et al. (2011) The inverse relationship between alanine aminotransferase in the normal range and adverse cardiovascular and non-cardiovascular outcomes. Int J Epidemiol 40: 1530–1538. dyr172 [pii];10.1093/ije/dyr172 [doi]. 10.1093/ije/dyr172 22158663

[12] Culleton BF, Manns BJ, Zhang J, Tonelli M, Klarenbach S, et al. (2006) Impact of anemia on hospitalization and mortality in older adults. Blood 107: 3841–3846. 2005-10-4308 [pii];10.1182/blood-2005-10-4308 [doi]. 10.1182/blood-2005-10-4308 16403909

[13] den Elzen WP, Willems JM, Westendorp RG, De Craen AJ, Assendelft WJ, et al. (2009) Effect of anemia and comorbidity on functional status and mortality in old age: results from the Leiden 85-plus Study. CMAJ 181: 151–157. cmaj.090040 [pii];10.1503/cmaj.090040 [doi]. 10.1503/cmaj.090040 PMC2717683 19635749

[14] van Bemmel T, Woittiez K, Blauw GJ, van der Sman-de Beer F, Dekker FW, et al. (2006) Prospective study of the effect of blood pressure on renal function in old age: the Leiden 85-Plus Study. J Am Soc Nephrol 17: 2561–2566. ASN.2005090902 [pii];10.1681/ASN.2005090902 [doi]. 10.1681/ASN.2005090902 16914542

[15] Novack V, Pencina M, Zahger D, Fuchs L, Nevzorov R, et al. (2010) Routine laboratory results and thirty day and one-year mortality risk following hospitalization with acute decompensated heart failure. PLoS One 5: e12184. 10.1371/journal.pone.0012184 [doi]. 10.1371/journal.pone.0012184 PMC2923147 20808904

[16] Bates CJ, Mansoor MA, Pentieva KD, Hamer M, Mishra GD (2010) Biochemical risk indices, including plasma homocysteine, that prospectively predict mortality in older British people: the National Diet and Nutrition Survey of People Aged 65 Years and Over. Br J Nutr 104: 893–899. S0007114510001236 [pii];10.1017/S0007114510001236 [doi]. 10.1017/S0007114510001236 PMC3445011 20398433

[17] May HT, Horne BD, Ronnow BS, Renlund DG, Muhlestein JB, et al. (2009) Superior predictive ability for death of a basic metabolic profile risk score. Am Heart J 157: 946–954. S0002-8703(09)00106-9 [pii];10.1016/j.ahj.2008.12.021 [doi]. 10.1016/j.ahj.2008.12.021 19376326

[18] Horne BD, May HT, Muhlestein JB, Ronnow BS, Lappe DL, et al. (2009) Exceptional mortality prediction by risk scores from common laboratory tests. Am J Med 122: 550–558. S0002-9343(09)00103-X [pii];10.1016/j.amjmed.2008.10.043 [doi]. 10.1016/j.amjmed.2008.10.043 19486718

[19] Giovannini S, Onder G, Liperoti R, Russo A, Carter C, et al. (2011) Interleukin-6, C-Reactive Protein, and Tumor Necrosis Factor-Alpha as Predictors of Mortality in Frail, Community-Living Elderly Individuals. J Am Geriatr Soc. 10.1111/j.1532-5415.2011.03570.x [doi]. 10.1111/j.1532-5415.2011.03570.x PMC4321727 21883115

[20] Pandya A, Weinstein MC, Gaziano TA (2011) A comparative assessment of non-laboratory-based versus commonly used laboratory-based cardiovascular disease risk scores in the NHANES III population. PLoS One 6: e20416. 10.1371/journal.pone.0020416 [doi];PONE-D-11-00842 [pii]. 10.1371/journal.pone.0020416 PMC3105026 21655241

[21] Studenski S, Perera S, Patel K, Rosano C, Faulkner K, et al. (2011) Gait speed and survival in older adults. JAMA 305: 50–58. 305/1/50 [pii];10.1001/jama.2010.1923 [doi]. 10.1001/jama.2010.1923 PMC3080184 21205966

[22] Taekema DG, Gussekloo J, Westendorp RG, De Craen AJ, Maier AB (2012) Predicting survival in oldest old people. Am J Med 125: 1188–1194. S0002-9343(12)00434-2 [pii];10.1016/j.amjmed.2012.01.034 [doi]. 10.1016/j.amjmed.2012.01.034 23017181

[23] Bootsma-van der Wiel A, Van Exel E, De Craen AJ, Gussekloo J, Lagaay AM, et al.. (2002) A high response is not essential to prevent selection bias: results from the Leiden 85-plus study. J Clin Epidemiol 55: 1119–1125. S089543560200505X [pii]. 10.1016/s0895-4356(02)00505-x 12507676

[24] Cockcroft DW, Gault MH (1976) Prediction of creatinine clearance from serum creatinine. Nephron 16: 31–41. 1244564 10.1159/000180580

[25] Weverling-Rijnsburger AW, Blauw GJ, Lagaay AM, Knook DL, Meinders AE et al.. (1997) Total cholesterol and risk of mortality in the oldest old. Lancet 350: 1119–1123. S0140673697044309 [pii]. 10.1016/s0140-6736(97)04430-9 9343498

[26] van Bemmel T, Gussekloo J, Westendorp RG, Blauw GJ (2006) In a population-based prospective study, no association between high blood pressure and mortality after age 85 years. J Hypertens 24: 287–292. 10.1097/01.hjh.0000200513.48441.8e [doi];00004872-200602000-00014 [pii]. 10.1097/01.hjh.0000200513.48441.8e 16508574

[27] Bloem BR, Haan J, Lagaay AM, van Beek W, Wintzen AR, et al (1992) Investigation of gait in elderly subjects over 88 years of age. J Geriatr Psychiatry Neurol 5: 78–84. 1590914 10.1177/002383099200500204

[28] Kempen GI, Miedema I, Ormel J, Molenaar W (1996) The assessment of disability with the Groningen Activity Restriction Scale. Conceptual framework and psychometric properties. Soc Sci Med 43: 1601–1610. S0277953696000573 [pii]. 10.1016/s0277-9536(96)00057-3 8961404

[29] Bootsma-van der Wiel A, Gussekloo J, De Craen AJ, Van Exel E, Knook DL et al.. (2001) Disability in the oldest old: “can do” or “do do”? J Am Geriatr Soc 49: 909–914. jgs49181 [pii]. 10.1046/j.1532-5415.2001.49181.x 11527482

[30] World Health Organisation (2007) International Classification of diseases and related disorders, 10th revision.

[31] Folstein MF, Folstein SE, McHugh PR (1975) “Mini-mental state”. A practical method for grading the cognitive state of patients for the clinician. J Psychiatr Res 12: 189–198. 0022–3956(75)90026–6 [pii]. 10.1016/0022-3956(75)90026-6 1202204

[32] Bootsma-van der Wiel A, De Craen AJ, Van Exel E, Macfarlane PW, Gussekloo J, et al (2005) Association between chronic diseases and disability in elderly subjects with low and high income: the Leiden 85-plus Study. Eur J Public Health 15: 494–497. 16014663 10.1093/eurpub/cki015

[33] Heeren TJ, Lagaay AM, von Beek WC, Rooymans HG, Hijmans W (1990) Reference values for the Mini-Mental State Examination (MMSE) in octo- and nonagenarians. J Am Geriatr Soc 38: 1093–1096. 2229862 10.1111/j.1532-5415.1990.tb01371.x

[34] von Elm E, Altman DG, Egger M, Pocock SJ, Gotzsche PC, et al. (2007) Strengthening the Reporting of Observational Studies in Epidemiology (STROBE) statement: guidelines for reporting observational studies. BMJ 335: 806–808. 335/7624/806 [pii];10.1136/bmj.39335.541782.AD [doi]. 10.1136/bmj.39335.541782.AD PMC2034723 17947786

[35] Gruenewald TL, Seeman TE, Karlamangla AS, Sarkisian CA (2009) Allostatic load and frailty in older adults. J Am Geriatr Soc 57: 1525–1531. JGS2389 [pii];10.1111/j.1532-5415.2009.02389.x [doi]. 10.1111/j.1532-5415.2009.02389.x PMC3650612 19682116

[36] Karlamangla AS, Singer BH, McEwen BS, Rowe JW, Seeman TE (2002) Allostatic load as a predictor of functional decline. MacArthur studies of successful aging. J Clin Epidemiol 55: 696–710. S0895435602003992 [pii]. 10.1016/s0895-4356(02)00399-2 12160918

[37] Seeman TE, McEwen BS, Rowe JW, Singer BH (2001) Allostatic load as a marker of cumulative biological risk: MacArthur studies of successful aging. Proc Natl Acad Sci U S A 98: 4770–4775. 10.1073/pnas.081072698 [doi];081072698 [pii]. 10.1073/pnas.081072698 PMC31909 11287659

[38] van Vliet P, Oleksik AM, van Heemst D, De Craen AJ, Westendorp RG (2010) Dynamics of traditional metabolic risk factors associate with specific causes of death in old age. J Gerontol A Biol Sci Med Sci 65: 488–494. glq014 [pii];10.1093/gerona/glq014 [doi]. 10.1093/gerona/glq014 20154178

[39] Lee SJ, Lindquist K, Segal MR, Covinsky KE (2006) Development and validation of a prognostic index for 4-year mortality in older adults. JAMA 295: 801–808. 295/7/801 [pii];10.1001/jama.295.7.801 [doi]. 10.1001/jama.295.7.801 16478903

[40] De Craen AJ, Westendorp RG (2006) Prognostic index for 4-year mortality in older adults. JAMA 296: 648–649. 296/6/648-a [pii];10.1001/jama.296.6.648-b [doi]. 10.1001/jama.296.6.648-b 16896101

[41] Christensen K, Thinggaard M, McGue M, Rexbye H, Hjelmborg JV, et al (2009) Perceived age as clinically useful biomarker of ageing: cohort study. BMJ 339: b5262. 20008378 10.1136/bmj.b5262 PMC2792675

[42] Christensen K, Iachina M, Rexbye H, Tomassini C, Frederiksen H, et al (2004) “Looking old for your age”: genetics and mortality. Epidemiology 15: 251–252. 15127920 10.1097/01.ede.0000112211.11416.a6

