# Correction: Predictive Value of a Profile of Routine Blood Measurements on Mortality in Older Persons in the General Population: The Leiden 85-Plus Study

**DOI:** 10.1371/journal.pone.0129985

**Published:** 2015-06-09

**Authors:** Anne H. van Houwelingen, Wendy P. J. den Elzen, Simon P. Mooijaart, Margot Heijmans, Jeanet W. Blom, Anton J. M. de Craen, Jacobijn Gussekloo

There is an error in the Table 2 legend. The values representing the 25th, 50th, and 75th percentile limits of Homocysteine laboratory results stratified for sex are incorrect. Please see the corrected Table 2 here.

**Table 2:** Univariate all-cause mortality risks for sex-dependent quartiles of laboratory results included in the laboratory profile (n = 562)

	Quartile of laboratory results				p for trend	P for 4th quartile compared to the other 3 quartilescombined
	1	2	3	4		
C-reactive protein	1 (ref)	1.10 (0.75–1.61)	1.36 (0.96–1.94)	2.11 (1.51–2.95	<0.001	<0.001
Homocysteine	1 (ref)	1.14 (0.76–1.71)	2.04 (1.40–2.60)	2.75 (1.92–3.96)	<0.001	<0.001
Hemoglobin*	1 (ref)	0.98 (0.67–1.42)	1.04 (0.72–1.48)	1.82 (1.31–2.54)	<0.001	<0.001
High density lipoprotein cholesterol*	1 (ref)	1.04 (0.72–1.50)	1.01 (0.70–1.47)	1.86 (1.33–2.60)	<0.001	<0.001
Alanine transaminase*	1 (ref)	1.00 (0.69–1.44)	0.88 (0.61–1.26)	1.72 (1.23–2.39)	0.005	0.004
Albumin *	1 (ref)	1.41 (0.94–2.19)	2.10 (1.45–3.06)	3.39 (2.33–4.95)	<0.001	<0.001
Creatinin clearance *	1 (ref)	0.96 (0.66–1.41)	1.32 (0.92–1.89)	1.85 (1.31–2.61)	<0.001	<0.001

Data represent hazard ratios and 95% confidence intervals, calculated with the univariate Cox-proportional hazard model

Laboratory results are divided into sex-dependent quartiles

25th, 50th and 75th percentile limits of laboratory results stratified for sex:

Hemoglobin: male 12.5–13.4–14.2 g/dL; female 12.0–12.8–13.6 g/dL

Hemoglobin: male 12.5–13.4–14.2 g/dL; female 12.0–12.8–13.6 g/dL

Alanine transaminase: male 11-15-20 U/L; female: 11-14-17 U/L

Albumin: male: 4.0–4.2–4.4 g/dL; female: 4.0–4.2–4.4 g/dL

Creatinin clearance: male: 39.4–47.2–53.9 ml/min; female: 36.8–43.4–50.8 ml/min

C-reactive protein: male: 2-4-8 mg/L; female: 1-4-8 mg/L

Homocysteine: male: 10.9–13.5–17.4 umol/L, female: 10.2–12.2–15.4 umol/L

* highest to lowest quartile
